# Artificial Intelligence Algorithm-Based Positron Emission Tomography (PET) and Magnetic Resonance Imaging (MRI) in the Treatment of Glioma Biopsy

**DOI:** 10.1155/2022/5411801

**Published:** 2022-03-23

**Authors:** Wei Wei, Liujia Ma, Liying Yang, Rong Lu, Cong Xi

**Affiliations:** ^1^Department of Neurosurgery, Affiliated Hospital of Yan'an University, Yan'an 716000, Shaanxi, China; ^2^Department of Neurology, Baoji Municipal People's Hospital, Baoji 722204, Shaanxi, China

## Abstract

This study was aimed at exploring the application value of positron emission tomography (PET) + magnetic resonance imaging (MRI) technology based on convolutional neural network (CNN) in the biopsy and treatment of intracranial glioma. 35 patients with preoperatively suspicious gliomas were selected as the research objects. Their imaging images were processed using CNN. They were performed with the preoperative head MRI, fluorodeoxyglucose (FDG) PET, and ethylcholine (FECH) PET scans to construct the cancer tissue contours. In addition, the performance of CNN was evaluated, and the postoperative pathology of patients was analyzed. The results suggested that the CNN-based PET + MRI technology showed a recognition accuracy of 97% for images. Semiquantitative analysis was adopted to analyze the standard uptake value (SUV). It was found that the SUV_FDG_ and SUV_FECH_ of grade II/III glioma were 9.77 ± 4.87 and 1.82 ± 0.50, respectively, and the SUV_FDG_ and SUV_FECH_ of grade IV glioma were 13.91 ± 1.83 and 3.65 ± 0.34, respectively. According to FDG PET, the mean value of SUV on the lesion side of grade IV glioma was greater than that of grade II-III glioma, and the difference was significant (*P* < 0.05), and similar results were obtained on FECH PET. It showed that CNN-based PET + MRI fusion technology can effectively improve the recognition effect of glioma, can more accurately determine the scope of glioma lesions, and can predict the degree of malignant glioma to a certain extent.

## 1. Introduction

Neuroglioma can be referred to as glioma for short. It is a relatively common malignant tumor of the central nervous system from neuroepithelium, accounting for about 40% of intracranial tumors. It is the most common primary intracranial tumor, affecting approximately 3 to 8 out of 100,000 people each year, affecting all ages with a higher occurrence in men [1, 2]. According to the mortality rankings released by the World Health Organization (WHO) in 1998, glioma is the second leading cause of death in tumor patients under the age of 34 and the third leading cause of death between the ages of 35 and 54. Its symptoms are mainly characterized by sudden headaches accompanied by projective vomiting and increased intracranial pressure such as papilledema and some localized nerve damage [3, 4]. Aggressive growth is the main growth pattern of gliomas, which leaves no clear demarcation point between them and the surrounding adjacent normal brain tissue. To a large extent, the clinical effect of surgical treatment and the prognosis of patients are determined by the degree of resection of cancer tissue [5].

At present, the diagnosis of glioma is mainly based on imaging methods, such as magnetic resonance imaging (MRI) and positron emission tomography (PET). MRI is currently the most widely used clinical imaging for diagnosing neurosurgical gliomas, and it can construct the outline of cancer tissue by enhancing the range of enhanced lesions in the scan. However, whether the blood-brain barrier is damaged is a key factor in determining whether MRI images are enhanced or not. This means that MRI may not fully reflect the extent of cancer tissue. For most low-grade gliomas and a small number of high-grade gliomas, MRI does not show enhancement or only partial enhancement, which makes MRI insurmountable in describing the outline of cancer tissue, especially for low-grade gliomas [6–8]. PET can diagnose lesions through the metabolic differences between normal and diseased tissues and use different molecular imaging agents to perform noninvasive, dynamic, qualitative, and quantitative analysis of the metabolism and proliferation of brain tumor tissue. Therefore, it can supplement important information to CT/MRI images, but there are obvious deficiencies in tissue identification and anatomical localization [9].

PET and MRI fusion technology can combine the metabolic and anatomical data displayed by PET and MRI, which makes the localization information in the treatment of clinical gliomas more comprehensive and the treatment effect is more optimized [10, 11]. PET + MRI image fusion shows important clinical value for early diagnosis of glioma, precise localization of lesions, disease-based symptomatic treatment, and comprehensive evaluation. However, a large amount of image data increases the workload of doctors, which easily leads to fatigue, missed diagnosis, and misdiagnosis of disease diagnosis and is also susceptible to the images of doctors' experience and knowledge level in the reading process, making the results highly subjective [12]. Therefore, artificial intelligence algorithm-assisted diagnosis shows important research significance and application value in accurately diagnosing lesions. Convolutional neural network (CNN), which combines deep learning technology, is a learning method that can simulate the brain level. It uses four methods, namely, local receptive field, weight sharing, subsampling, and sparse connection, to process two-dimensional images. In addition, the advantage of CNN is that it can directly identify and feature the input original image, omitting the tedious process of preprocessing it, so CNN has extraordinary achievements in pattern recognition [13–15].

Taking glioma PET + MRI images as the research objects, this work constructed an artificial intelligence-based CNN auxiliary diagnosis model. The model was used in the classification and identification of gliomas to help clinicians make rapid diagnosis and treatment and to achieve the consistency of images and clinical research speculations. It aimed to promote the process of artificial intelligence, deepen its application in the medical field, and provide a certain theoretical basis for the diagnosis and treatment of neurosurgical gliomas in clinical practice.

## 2. Materials and Methodologies

### 2.1. General Data

From March 2018 to January 2020, 35 patients with highly suspected gliomas before surgery were treated in hospital. The general data about these patients is male in 23 cases and female in 12 cases. The age is from 23 to 67, with an average age of 45.3. The length of disease course is from 1 to 4 years. The main clinical manifestations of the patients were sudden headache with occasional projectile vomiting, general malaise, slurred speech, and seizures. The patients and their families had fully understood the situation and signed the informed consent forms, and this study had been approved by the medical ethics committee of the hospital.

Inclusion criteria were as follows: (i) patients whose diseases met the diagnostic criteria for glioma, which could be proved by definite pathological results of surgery or stereotactic needle biopsy; (ii) all patients who were first onset; (iii) patients with complete imaging data; and (iv) all patients who were aware of the study content and signed the informed consents.

Exclusion criteria were as follows: (i) patients with a history of brain space-occupying lesions; (ii) patients with a history of glioma treatment; and (iii) patients with a history of head trauma.

### 2.2. Preoperative Imaging Examination

PET scanning: all patients were performed with the examination 2 to 3 days before operation. The tracer 11C-Met 550–750 mBq was injected intravenously 6 hours after meal. After 20 minutes, PET was adopted for scanning, and cross sections, coronal planes, and sagittal planes were displayed, respectively.

MRI scanning: all patients were performed with MRI scanning on heads 1 day before the operation to determine the size of tumors. Conventional T1-weighted image (T1WI) and T2WI series examinations were implemented, respectively, by the 1.5 T intraoperative scanner. After gadopentetate dimeglumine (Gd-DTPA) was injected intravenously, T1WI enhancement scanning (T1-Gd) was conducted. In addition, some patients received magnetic resonance angiography (MRA) and diffusion tensor imaging (DTI). The grading criteria for gliomas in imaging examinations could be summarized as follows: for low-grade glioma (grade II), the diffuse astrocytoma showed relatively uniform signal, low signal on T1W, mostly no enhancement, and high signal on T2W and FLAIR. For, anaplastic glioma (grade III), when astrocytoma or oligodendrocytoma considered by MRI was enhanced, it indicated a high possibility of anaplastic. For glioma in grade IV, the main features of glioblastoma were irregular peripheral enhancement and massive central necrosis, and brain edema was visible outside the enhancement. Gliosarcoma, due to the predominance of sarcoma or glioma components, presented a real heterogeneously enhancing mass or a glioblastoma-like appearance, respectively.

### 2.3. Assessment of Uptake of PET Tracers

Visual analysis was adopted in the assessment. Combined with the lesion ranges of MRI images, the uptake of imaging agents was divided into three components as follows [16]:

1. Low metabolism with uptake being lower or similar to white matters
2. Intermediate metabolism with uptake being higher than white matters while significantly less than gray matters
3. High metabolism with uptake being similar, equal to, or higher than gray matters

Apart from the concentration of imaging agents within lesion ranges, the assessment of uptake needed to be combined with the distribution forms and uniformity of imaging agents and the clear state of boundaries.

### 2.4. Preparation of CNN Model Structure

#### 2.4.1. Convolutional Layer

As the core of the extraction of features in a convolutional neural network, the hidden layer has two special structures, including convolutional layer and subsampling layer. Local features of particular areas in local receptive fields are extracted by the convolutional layer. To be specific, a learnable kernel is convolved with the feature image on each layer to output the feature image on the next layer by the activated function. The convolution expression is shown in the following equation:

(1) $$\begin{array}{c} x_{a}^{l}=f\left(\sum_{i\in Ma}x_{a}^{l-1}•k_{ia}^{l}+c_{a}^{l}\right). \end{array}$$

In the above equation, *l* represents the number of layers, *k* stands for convolution kernel, *x*_*a*_^*l*−1^ means a feature image on the upper layer, *k*_*ia*_^*l*^ refers to the weight of convolution kernel, *c* means the offset item of each output feature image, and f() refers to the activated function. The incomplete connection mode is adopted in CNN, and this mode is featured with the sparse link between the neurons on upper and lower levels by local spatial correlation among layers. Hence, every output feature image may contain the convolution of multiple input images, which function differently due to the discrepancies of weights in convolution kernels.

#### 2.4.2. Subsampling

Pooling calculation is performed for input by subsampling layer, and the feature dimension as well as the resolution can be reduced without repeated sampling. The number of input feature images and output feature images obtained by subsampling calculation are both *n*, but the original image is 2 times as large as the output feature images acquired by subsampling. The subsampling layer is expressed as

(2) $$\begin{array}{c} x_{a}^{l}=f\left(\beta_{a}^{l}\text{down}\left(x_{a}^{l-1}\right)+c_{a}^{l}\right). \end{array}$$

In the above equation, down (•) means subsampling function, *β* stands for multiplicative deviation, and *c* refers to additive deviation. After the features of images are obtained by using convolution, the classifier cannot categorize features directly because of large amounts of calculation, excessive time consumption, and easy fitting. To reduce the size of feature images, the subsampling layer is connected after the convolution layer. The neutrons input by the pooling layer is on the upper sampling window convolution layer in which neutrons gather form the value of neutrons. The value usually contains mean sampling and maximum pool sampling. The pooling results in a significant decrease of the number of neutrons in the model and shows the robustness in the horizontal movement and transformation of the input space.

#### 2.4.3. Fully Connected Layer

In fully connected layers, all feature images (two-dimensional images), which are connected to the one-dimensional features, are used as the input to the fully connected network. The output of the fully connected layer is acquired by the input weighed sum and the response of the activated function.

(3) $$\begin{array}{c} x^{l}=f\left(v^{l}x^{l-1}+c^{l}\right). \end{array}$$

In the above equation, *v*^*l*^ represents the weight coefficient of the fully connected network, *x*^*l*−1^ refers to feature images, and *c*^*l*^ means the offset items of the fully connected layer.

### 2.5. Softmax Classifier

Logical regression is the basis of the expansion of the Softmax regression classifier, and the training sample set of Softmax consists of *m* labelled samples: {*x*^(1)^, *y*^(1)^, *x*^(2)^, *y*^(2)^,…, *x*^(*m*)^, *y*^(*m*)^}. Among these elements, x^(i)^ stands for input features and *x*^(*i*)^ ∈ *S*^*n*+1^, *n* + 1 is the dimension of eigenvector *x*, *y*^(*i*)^ means classification labels, and logistic regression label is set as *y*^(*i*)^ ∈ {0,1}.

If the loss function is expressed as (4), the cost function of the minimization of the parameter *θ* is expressed as (5).

(4) $$\begin{array}{c} p_{\theta }\left(x\right)=\frac{1}{1+\exp\left(-\theta^{T}x\right)}, \end{array}$$

(5) $$\begin{array}{c} A\left(\theta \right)=-\frac{1}{m}\left[\sum_{i-1}^{m}y^{i}\log\;p_{\theta }\left(x^{i}\right)+\left(1-y^{i}\right)\log\left(1-p_{\theta }\left(x^{\left(i\right)}\right)\right)\right]. \end{array}$$

In the training samples of Softmax regression, different types of samples are denoted by *a*, the probability value of *a* is estimated by the function *p*_*θ*_(*x*), and the probability value is shown as *q*(*y*=*a|x*). *p*_*θ*_(*x*) is presented as follows:

(6) $$\begin{array}{c} {\text{p}}_{\theta }\left({\text{x}}^{\text{i}}\right)=\left[\begin{array}{c} \text{q}\left({\text{y}}^{\text{i}}=1|{\text{x}}^{\left(\text{i}\right)};\theta \right)\\ \text{q}\left({\text{y}}^{\text{i}}=2|{\text{x}}^{\left(\text{i}\right)};\theta \right)\\ \text{q}\left({\text{y}}^{\text{i}}=\text{k}|{\text{x}}^{\left(\text{i}\right)};\theta \right) \end{array}\right]=\frac{1}{\sum_{\text{a}=1}^{\text{k}}{\text{e}}^{\theta_{\text{a}}^{T}{\text{x}}^{\left(\text{i}\right)}}}\left[\begin{array}{c} {\text{e}}^{\theta_{1}^{T}{\text{x}}^{\left(\text{i}\right)}}\\ {\text{e}}^{\theta_{2}^{T}{\text{x}}^{\left(\text{i}\right)}}\\ {\text{e}}^{\theta_{\text{k}}^{T}{\text{x}}^{\left(\text{i}\right)}} \end{array}\right]. \end{array}$$

In the above equation, *θ*_1_,  *θ*_2_,…, *θ*_*k*_ are parameters of the model and *θ*_1_, *θ*_2_, ···, *θ*_k_ ∈ *S*^n+1^. In the process of *Soft*max regression, *θ* is written in the column matrix as $\theta =\left[\begin{array}{c} \theta_{1}^{T}\\ \theta_{2}^{T}\\ \theta_{\text{k}}^{T} \end{array}\right]$.

Based on the logistic regression, *Soft*max regression cost function is analyzed. In the function, 1 represents indicative function. 1{the expression value is true} = 1, and 1{the expression value is false} = 0, which demonstrate that the equation of cost function is expressed as follows:

(7) $$\begin{array}{c} A\left(\theta \right)=-\frac{1}{\text{m}}\left[\sum_{\text{i}=1}^{\text{m}}\sum_{\text{a}=0}^{1}1\left\{{\text{y}}^{\left(\text{i}\right)}\left.=\text{a}\right\}\log\text{q}\left({\text{y}}^{\left(\text{i}\right)}=\text{a}|{\text{x}}^{\left(\text{i}\right)};\theta \right)\right.\right]. \end{array}$$

The cost function of *Soft*max is shown as follows:

(8) $$\begin{array}{c} A\left(\theta \right)=-\frac{1}{\text{m}}\left[\sum_{\text{i}=1}^{\text{m}}\sum_{\text{a}=1}^{\text{k}}1\left\{{\text{a}}^{\left(\text{i}\right)}\left.=\text{a}\right\}\log\frac{{\text{e}}^{\theta_{\text{a}}^{T}{\text{x}}^{\left(\text{i}\right)}}}{\sum_{\text{l}=1}^{\text{k}}{\text{e}}^{\theta_{\text{l}}^{T}{\text{x}}^{\left(\text{i}\right)}}}\right.\right]. \end{array}$$

The commonly used method of minimizing cost function is iterative optimization algorithm. The partial derivative of *θ*_a_ by *A*(*θ*) is shown as follows:

(9) $$\begin{array}{c} \frac{\partial A\left(\theta \right)}{\partial \theta_{\text{al}}}=\nabla_{\theta_{\text{a}}}A\left(\theta \right)=-\frac{1}{\text{m}}\sum_{\text{i}=1}^{\text{m}}\left[{\text{x}}^{\left(\text{i}\right)}\left(1\left\{{\text{y}}^{\left(\text{i}\right)}=\left.\text{a}\right\}-\text{q}\left({\text{y}}^{\left(\text{i}\right)}=\text{a}|{\text{x}}^{\left(\text{i}\right)},\theta \right)\right.\right)\right]. \end{array}$$

Later, the cost function *A*(*θ*) is minimized by the gradient descent algorithm. The gradient descent means the update of the parameter *θ* along with every iteration, which is shown as *θ*_a_=*θ*_a_ − z∇_*θ*a_*A*(*θ*)(a=1,2, ···, k).

To avoid overfitting, a regularization item *λ*/2∑_i=1_^k^∑_a=0_^n^*θ*_ia_^2^ is added behind the cost function and the excessively large parameter values are penalized, and then the equation for the regression cost function is derived, which is shown as follows:

(10) $$\begin{array}{c} A\left(\theta \right)=-\frac{1}{\text{m}}\left[\sum_{\text{i}=1}^{\text{m}}\sum_{\text{a}=1}^{\text{k}}1\left\{{\text{a}}^{\left(\text{i}\right)}=\left.\text{a}\right\}\log\frac{{\text{e}}^{\theta_{\text{a}}^{T}{\text{x}}^{\left(\text{i}\right)}}}{\sum_{\text{l}=1}^{\text{k}}{\text{e}}^{\theta_{\text{l}}^{T}{\text{x}}^{\left(\text{i}\right)}}}\right.\right]+\frac{\lambda }{2}\sum_{\text{i}=1}^{\text{k}}\sum_{\text{a}=0}^{\text{n}}\theta_{\text{ia}}^{2}. \end{array}$$

To achieve the *Soft*max regression and classification, the derivative of the minimized cost function is taken and shown as follows:

(11) $$\begin{array}{c} \frac{\partial A\left(\theta \right)}{\partial \theta_{\text{al}}}=\nabla_{\theta_{\text{a}}}A\left(\theta \right)=-\frac{1}{\text{m}}\sum_{\text{i}=1}^{\text{m}}\left[{\text{x}}^{\left(\text{i}\right)}\left(1\left\{{\text{y}}^{\left(\text{i}\right)}=\text{a}\right\}-\text{q}\left({\text{y}}^{\left(\text{i}\right)}=\text{a}|{\text{x}}^{\left(\text{i}\right)};\theta \right)\right)\right]+\lambda \theta_{\text{a}}. \end{array}$$

At the end, the Softmax regression classification model is generated by the minimization cost equation *A*(*θ*).

In the successive operations of convolutional neural networks and subsampling, the advantage of convolutional neural network in horizontal movement, scaling, and invariance in rotation as well as distortion is local receptive field, weight sharing, subsampling, and sparsity connection. While the convolution layer is connected to the neurons in a small neighborhood, weight sharing reduces the weight parameter obviously. The dimension of the subsampling features lowers, and the sparsity connection makes the network less complex, which enhances the generalization capacity and robustness of convolutional neural network in image comprehension.

### 2.6. Image Fusion

The 3D scan data of MRI and PET in DICOM format were imported into the system graphics workstation. The registration program automatically fused MRI T1-enhanced images, FDG PET images, and FECH PET images into the system. The accuracy of fusion was detected by craniofacial landmarks such as the nasal tip and inner conjunct, and manual fine-tuning can be performed if necessary. The fused images were still displayed in the form of MRI T1-enhanced images, FDG PET images, and FECH PET images, respectively, and lateral, sagittal, and coronal images can be displayed simultaneously as needed.

### 2.7. Formulation and Implementation of Operation

Formulation of operation: on the images of MRI (T1-Gd or T2WI) and 11C-Met PET cross sections, the contours of tumors were sketched, respectively. Concerning the cases whose lesions on the T1-Gd series were significantly enhanced, enhancement areas were regarded as tumor lesions. If the series did not enhance imaging or enhance it into punctiform shapes so that the contours of tumors could not be sketched, high signal areas of the T2WI series were viewed as tumor lesions. The areas where the uptake of 11C-Met PET images was obviously greater than peripheral normal gray matters were adopted as tumor lesions. When the contours of tumors were plotted, subjective visual manual drawing was utilized combined with the segmentation program and according to the image gray scale automatic plotting method. By the above methods, the contours of lesions in two images and the boundaries between intersection areas were sketched, respectively. The contributions of two different images in tumor sketch were described by the percentage (discrepancy-PET, %) of the volume of nonintersecting areas of PET and MRI images in that of the lesions shown by PET and the percentage (discrepancy-PET, %) of the volume of nonintersecting areas of MRI images and PET in that of the lesions shown by MRI, respectively.

Implementation of operation: the operation was implemented according to the postoperative operation plan and the relationship between tumors and normal brain tissues under the navigation microscope. Tumor excision was based on 11C-Met PET images or MRI images. To avoid brain tissue drift, intraoperative MRI scanning was performed on the cases with large intraoperative tumors and unclear boundaries between tumors and normal brain tissues hinted by the microscope. Besides, images were imported into the neurological system's computer workstation. After that, the same method was adopted to fuse and compare intraoperative MRI results with preoperative images to determine tumor excision.

### 2.8. Statistical Analysis

SPSS 23.0 software was used for statistical analysis of all data, measurement data were analyzed using independent samples *t*-test, count data were expressed as percentage, and *χ*2 test was used.

## 3. Results

### 3.1. Comparison of Performance of Convolutional Neural Networks

**Figure 1:**
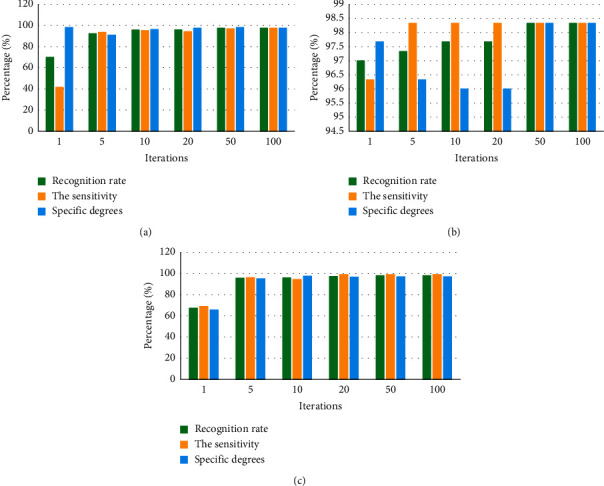
Comparison on the performance of CNNs. The performance evaluation results of (a) PET-CNN, (b) MRI-CNN, and (c) PET/MRI-CNN.

The parameter migration method was adopted to construct convolutional neural networks, and intracranial glioma with different modes of PET, MRI, and PET + MRI was identified.  Figure 1(a), Figure 1(b), and Figure 1(c) show the evaluation results of performance of PET-CNN, MRI-CNN, and PET/MRI-CNN, respectively.

According to the above figures, the recognition accuracy of PET-CNN reached nearly 95% after the iteration occurred more than 10 times. Without the growing iteration numbers, accuracy was increased slightly, and it amounted to 97.67% after 50 iterations. In general, sensitivity was slightly lower than specific degree. In contrast, the recognition rate of MRI-CNN was higher with recognition accuracy reaching about 97%. After 10 iterations, it tended to be stable without being enhanced by the increasing iteration numbers. The recognition rate of fused PET/MRI images by PET/MRI-CNN reached 97%, while the specific degree was relatively low.

### 3.2. Imaging Performance

**Figure 2:**
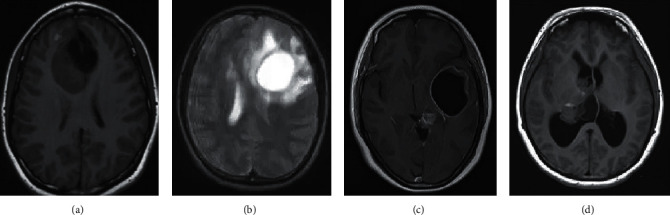
MRI images of patients with glioma in grade IV. The patient was a female, 62 years old. (a, b) MRI plain scan images and (c, d)MRI enhanced images.

**Figure 3:**
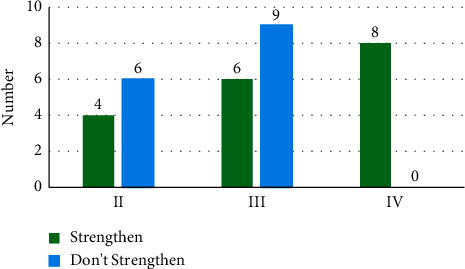
Enhancement of gliomas of different grades by MRI.

**Figure 4:**
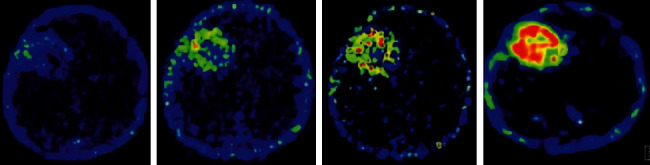
PET images of glioma patients. From left to right, they were the images of 1 h, 8 h, 12 h, and 24 h of injection of tracer, respectively.

**Figure 5:**
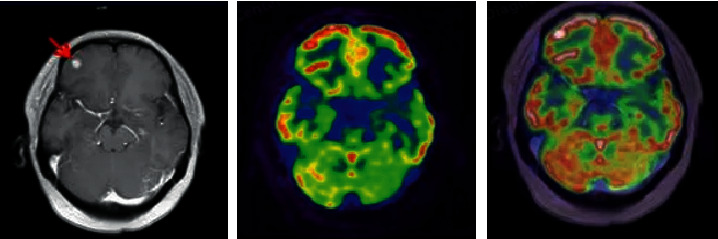
PET + MRI fusion images of glioma patients. From left to right, they were the fusion images and the images at 8 h and 24 h after the tracer injection. The arrows indicate the lesions.

MRI of all patients showed T1 and T2 signals with different lengths. The enhancement scan demonstrated enhancement in 19 cases, and no obvious enhancement was detected in 16 cases. According to the type of pathology, enhancement was found in 4 cases with glioma of grade II and 6 cases with glioma of grade III. Enhancement was shown in all eight cases with glioblastoma multiforme, and obvious inhomogeneous enhancement was demonstrated in the other two patients with central nervous system vasculitis. Figures 2 and 3 show specific details.  Figure 4 shows the PET images obtained after tracer injection in a 45-year-old male glioma patient.  Figure 5 shows the PET + MRI fusion images of a 54-year-old female glioma patient after tracer injection.

**Figure 6:**
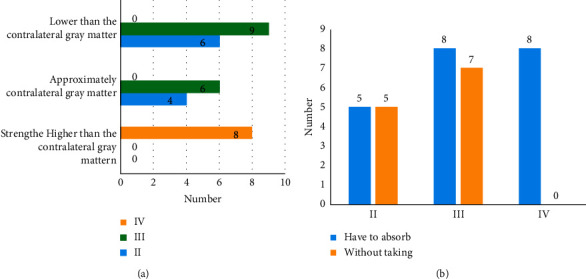
Uptake of tracers by gliomas of different grades. (a) The uptake of 18F-FDG and (b) the uptake of 18F-FECH.

On PDG PET, eight cases with the uptake greater than the contralateral gray matter were all patients with glioblastoma multiforme. Among 12 cases with the uptake close to the contralateral gray matter, six were patients with glioma of grade III, four were diagnosed with glioma of grade II, and 2 were patients with central nervous system vasculitis. Among 15 cases with the uptake less than the contralateral gray matter but greater than the contralateral white matter, nine were patients with glioma of grade III and six were diagnosed with glioma of grade II. On 18F-FECH PET, there were 21 cases with the uptake greater than background brain and 12 cases without obvious uptake. Obvious uptake of 11C-MET was observed in all eight cases with glioblastoma multiforme. Among 12 cases without uptake, five were patients with glioma of grade II and seven had glioma of grade III. Detailed information is shown in Figures 6(a) and 6(b).

**Figure 7:**
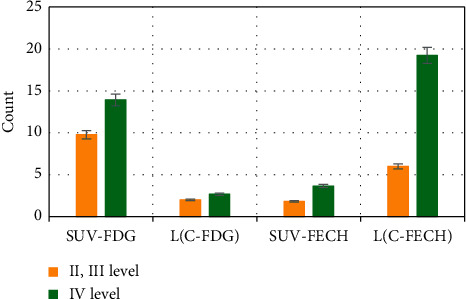
Semiquantitative analysis of the uptake of tracers by gliomas of different grades.

After measurement, it was found that the SUVFDG and SUVFECH of grade II/III glioma were 9.77 ± 4.87 and 1.82 ± 0.50, respectively, and the SUVFDG and SUVFECH of grade IV glioma were 13.91 ± 1.83 and 3.65 ± 0.34, respectively. The L/CFDG and L/CFECH values of grade II/III glioma were 2.00 ± 0.34 and 5.98 ± 3.88, respectively; and the L/CFDG and L/CFECH values of grade IV glioma were 2.68 ± 0.10 and 19.21 ± 6.30, respectively. See Figure 7 for details.

### 3.3. Construction of Contours of Cancer Tissues

According to the positional relationship of the contour lines of lesions on PDG PET and MRI, lesions were divided into the following six categories. (I) PET lesions within MRI lesions; (II) the contour lines of PET lesions and MRI lesions do not cross, but may overlap or not; (III) the contour lines of MRI lesions within PET lesions; (IV) the contour lines of PET and MRI lesions are roughly equal; (V) the contour lines of MRI are vague, so only PET lesions are detectable; and (VI) the contour lines of PET are vague, so only MRI lesions are detectable.

**Figure 8:**
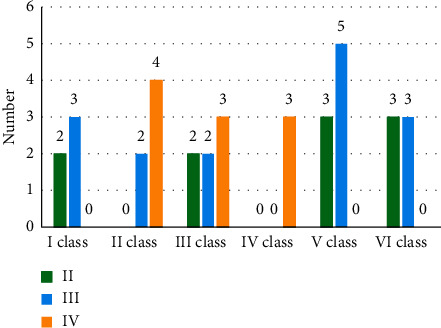
Classification of the manifestations of glioma of different grades on PDG PET.

The number of gliomas in grades II, III, and IV was 2, 3, and 0 in class I; it was 0, 2, and 4 in class II, and it was 2 and 2 in class III. The number of cases in category IV was 0, 0, and 3, respectively. The number of cases in category V was 3, 5, and 0, and the number of cases in category VI was 3, 3, and 0, respectively (as shown in Figure 8).

**Figure 9:**
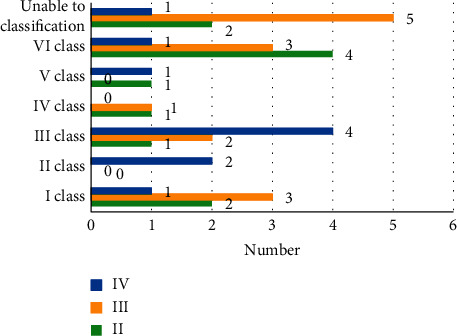
Classification of the manifestations of gliomas of different grades on FECH PET.

Classification of the positional relationship of lesions on 18F-FECH PET and MRI was given as follows (as illustrated in Figure 9). The number of grade II, III, and IV gliomas were 2, 3, and 1 in class I, 0, 0, and 2 in class II, and 1 and 2 in class III. There are 1, 1, and 0 cases in category IV, 1, 0, and 1 in category V, and 4, 3, and 1 in category VI. The number of cases classified was 2, 5, and 1, respectively.

## 4. Discussion

The prerequisite for the embodiment of its one value of convolutional neural network is accurate imaging data. Conventional MRI imaging is the most applied imaging technique so far. The Gd-DTPA enhanced sequence is often applied in the construction of cancer tissues. However, enhancement is not shown on MRI or can be observed on only a few MRIs since blood-brain barriers are not impaired completely in most of gliomas of lower grade and a few gliomas of higher grade. It is hard to distinguish cancer tissues from pericancerous edema merely by normal T1WI or T2WI [17, 18]. In the research, it was found that the recognition rate of fused PET/MRI images by PET/MRI-CNN reached 97%. Because fused images themselves could enhance the discernibility degree of lesion areas, make images clearer as well as significant visual effects, and show lesion ranges more obviously, the recognition effects of glioma in neurosurgery by convolutional neural networks were enhanced. However, the specific degree of the networks was relatively low, which indicated a high misdiagnosis rate.

PET is a metabolic imaging method with which noninvasive, dynamic, qualitative, and quantitative analysis of the metabolism and proliferation of cancer tissues can be performed based on different molecular imaging agents. The use of this technique is an indispensable supplement to the presentation of anatomic information by CT/MRI. Metabolic and anatomic data provided by PET and MRI, respectively, can be combined effectively by the joint use of PET and MRI, which provides more complete location information and qualitative information for the clinical treatment of glioma to optimize therapeutic effects [19]. Kebir et al. (2019) [20] performed surgical treatment on patients with gliomas of different grades with the navigation of 11C-MET PET fused with MRI. Without the enhanced MRI, PET tracers can be used to excise cancer tissues with high malignancy in locally concentrated areas. Hara et al. (2020) [21] made a statistical comparison of the tumor excision rate and the prognosis of patients between the PET/MRI navigation system and a single MRI navigation system, and 11C-MET was used as PET tracer. Totally, 36 surgeries were performed on 33 patients, 17 of which were navigated by PET/MRI and 19 were navigated by MRI. The results showed that PET/MRI could offer more information about the location of tumors, but there was no significant difference between the complications of the two surgeries. The total excision rate of the PET/MRI navigation system was higher than that of the MRI navigation system, and the PET/MRI navigation system survived longer than the MRI navigation system.

In this research, PET and MRI fusion technology was applied before surgeries to determine if biopsy or surgery should be performed on each of 35 patients suspected of glioma according to the location of lesions and imaging manifestations. The analysis of the manifestations of each case on MRI showed that glioblastoma multiforme in eight cases were all enhanced, but the enhancement of glioma could be observed only in 10 out of 25 cases with grades II-III, which proved that MRI enhancement could predict the severity of cancer tissues to some degree. However, the prediction was indirect because the judgement on if the blood-brain barrier was intact was necessary and closely related. This opinion was consistent with related literature reports [22, 23]. Meanwhile, eight cases with the uptake greater than the gray matter were all glioblastoma multiforme patients, and those with the uptake close to or less than gray matter were all diagnosed with glioma of grades II-III. In terms of the uptake of 18F-FECH (tracers), the obvious uptake of 18F-FECH was detected in all eight cases with glioblastoma multiforme, and the WHO grade of 12 glioma cases without obvious uptake were all II-III. According to the ratio of the standard uptake value of the lesions on FDG PET and the standard uptake value of lesions and contralateral brain tissues in semiquantitative analysis, the average of gliomas of grades II-III was lower than that of glioblastoma multiforme. These results were similar to the data obtained from FECH PET. Based on these analyses, it is shown that patients with the higher WHO grade of glioma probably means the higher uptake of two types of tracers. Unfortunately, the number of cases included in this research is small, and the analysis is not tested by statistics. The research shows that the PET uptake of tracers can better reflect the severity of glioma, which is consistent with some previous reports [24, 25].

In the comparison of the contours of lesions on PET and MRI, five PET lesions were smaller than those on MRI and seven PET lesions were larger than those on MRI on FDG PET. The result indicated that the range of lesions shown on PET was inclined to become larger than that presented by MRI. On FECH PET, tumor contours could not be determined by MRI and PET in eight cases, which showed the supplementary information provided for the cases with different tumor contours determined on MRI was not ideal. Nonetheless, a conclusion with statistical meaning could not be drawn because of the small number of cases. Katsanos et al. (2019) [26] reported that the range of lesions was smaller than that of lesions on MRI in nearly 85% of cases examined by FDG PET. The results showed an inconsistency with the results of the observation in this research. The appearance of the discrepancy may be related to different operating conditions of PET and the differences in subjective descriptions of constructing the contours of cancer tissues.

## 5. Conclusion

The PET + MRI fusion technology based on CNN can make the image clearer and the visual sense stronger. At the same time, it can improve the recognition effect of neurosurgical glioma and more accurately determine the scope of glioma lesions. In the absence of enhancement, there was a significant advantage especially in low-grade gliomas. However, due to the limited sample size, it lacked overall representativeness. In the future, it will increase the number of samples and conduct more in-depth studies on gliomas.

## Data Availability

The data used to support the findings of this study are available from the corresponding author upon request.
